# Increased cranio-caudal spinal cord oscillations are the cardinal pathophysiological change in degenerative cervical myelopathy

**DOI:** 10.3389/fneur.2023.1217526

**Published:** 2023-11-08

**Authors:** Nikolai Pfender, Jan Rosner, Carl M. Zipser, Susanne Friedl, Martin Schubert, Reto Sutter, Markus Klarhoefer, José M. Spirig, Michael Betz, Patrick Freund, Mazda Farshad, Armin Curt, Markus Hupp

**Affiliations:** ^1^Spinal Cord Injury Center, Balgrist University Hospital, Zurich, Switzerland; ^2^University Spine Center Zurich, Balgrist University Hospital, Zurich, Switzerland; ^3^Department of Neurology, University Hospital Bern, Inselspital, Bern, Switzerland; ^4^Radiology, Balgrist University Hospital, Zurich, Switzerland; ^5^Siemens Healthineers AG, Zurich, Switzerland

**Keywords:** spinal cord motion, spinal cord oscillations, spinal stenosis, degenerative cervical myelopathy, phase contrast MRI

## Abstract

**Introduction:**

Degenerative cervical myelopathy (DCM) is the most common cause of non-traumatic incomplete spinal cord injury, but its pathophysiology is poorly understood. As spinal cord compression observed in standard MRI often fails to explain a patient's status, new diagnostic techniques to assess DCM are one of the research priorities. Minor cardiac-related cranio-caudal oscillations of the cervical spinal cord are observed by phase-contrast MRI (PC-MRI) in healthy controls (HCs), while they become pathologically increased in patients suffering from degenerative cervical myelopathy. Whether transversal oscillations (i.e., anterior–posterior and right–left) also change in DCM patients is not known.

**Methods:**

We assessed spinal cord motion simultaneously in all three spatial directions (i.e., cranio-caudal, anterior–posterior, and right–left) using sagittal PC-MRI and compared physiological oscillations in 18 HCs to pathological changes in 72 DCM patients with spinal canal stenosis. The parameter of interest was the amplitude of the velocity signal (i.e., maximum positive to maximum negative peak) during the cardiac cycle.

**Results:**

Most patients suffered from mild DCM (mJOA score 16 (14–18) points), and the majority (68.1%) presented with multisegmental stenosis. The spinal canal was considerably constricted in DCM patients in all segments compared to HCs. Under physiological conditions in HCs, the cervical spinal cord oscillates in the cranio-caudal and anterior–posterior directions, while right–left motion was marginal [e.g., segment C5 amplitudes: cranio-caudal: 0.40 (0.27–0.48) cm/s; anterior–posterior: 0.18 (0.16–0.29) cm/s; right–left: 0.10 (0.08–0.13) cm/s]. Compared to HCs, DCM patients presented with considerably increased cranio-caudal oscillations due to the cardinal pathophysiologic change in non-stenotic [e.g., segment C5 amplitudes: 0.79 (0.49–1.32) cm/s] and stenotic segments [.g., segment C5 amplitudes: 0.99 (0.69–1.42) cm/s]). In contrast, right–left [e.g., segment C5 amplitudes: non-stenotic segment: 0.20 (0.13–0.32) cm/s; stenotic segment: 0.11 (0.09–0.18) cm/s] and anterior–posterior oscillations [e.g., segment C5 amplitudes: non-stenotic segment: 0.26 (0.15–0.45) cm/s; stenotic segment: 0.11 (0.09–0.18) cm/s] remained on low magnitudes comparable to HCs.

**Conclusion:**

Increased cranio-caudal oscillations of the cervical cord are the cardinal pathophysiologic change and can be quantified using PC-MRI in DCM patients. This study addresses spinal cord oscillations as a relevant biomarker reflecting dynamic mechanical cord stress in DCM patients, potentially contributing to a loss of function.

## 1. Introduction

Degenerative cervical myelopathy (DCM) is the most common cause of non-traumatic incomplete spinal cord injury (1, 2). As spinal cord compression observed in standard MRI often fails to explain a patient's status (3–6), new diagnostic techniques to assess DCM are one of the research priorities (7). The spinal cord is subject to cardiac-related oscillations, which were initially shown by intraoperative ultrasound (8). Later on, phase contrast MRI (PC-MRI) revealed increased cranio-caudal spinal cord oscillations at the level of cervical spinal stenosis and also in adjacent segments in DCM patients (9–12). The highest increase in cranio-caudal oscillations was observed at the site of the cervical stenosis, suggesting that this is a causal mechanism resulting in excessive strain on the entire cervical cord through stretch and compression of adjacent segments (13). Additionally, in contrast to a physiological resting phase in healthy conditions, an altered motion pattern with restless oscillations of the spinal cord throughout the cardiac cycle in DCM patients was observed (14). Assuming ~100,000 heartbeats and subsequent oscillations per day, dynamic mechanical stress on the spinal cord tissue may be underestimated in DCM pathophysiology. Supporting this hypothesis, increased cranio-caudal spinal cord motion was associated with sensory deficits (9, 12) and impaired electrophysiological readouts (11) in DCM patients. Thus, increased cranio-caudal spinal cord motion has emerged as a new and promising diagnostic biomarker in DCM patients, reflecting dynamic mechanical stress on the spinal cord. Therefore, studies on cervical spinal cord motion in DCM patients focused on oscillations in the cranio-caudal direction only, while relevant anterior–posterior and right–left oscillations of the cord in patients suffering from spine metastasis were previously observed (15). In this study, we aimed to investigate cervical spinal cord motion simultaneously in all three spatial directions (i.e., cranio-caudal, anterior–posterior, and right–left) under physiological conditions in healthy controls (HCs) in comparison to its pathologic changes in DCM patients. We hypothesize that, compared to HCs, anterior–posterior and right–left spinal cord oscillations will be reduced in DCM patients due to space constraints at the cervical stenosis level and that the cranio-caudal component of the oscillations is enhanced, leading to increased dynamic mechanical stress inflicted upon the spinal cord.

## 2. Methods

### 2.1. Population

This prospective, cross-sectional study recruited 72 DCM patients from the outpatient clinic of the University Hospital Balgrist, Zurich, Switzerland between September 2018 and June 2021. The population was in part reported previously (14, 16), and the findings presented here were not reported earlier. The inclusion criteria were as follows: cervical spinal stenosis on T2-weighted (T2-w) MRI, clinical symptoms and signs consistent with degenerative cervical myelopathy (17) (i.e., pain, sensory or motor deterioration in the upper or lower limbs, and gait or bladder dysfunction), and age 18–80 years. Other neurological diseases (e.g., radiculopathy at the lower limbs, polyneuropathy, and CNS disorders) were excluded upon extensive examination (e.g., cranial MRI and electrophysiologic examinations) prior to study inclusion. The exclusion criteria were general MRI contraindications (e.g., pacemaker), epileptic seizures, mental illness, severe medical illness, and pregnancy. A previously reported cohort of 18 HCs (18) was used for the evaluation of cervical spinal cord motion under physiological conditions and for comparison to patients. For HCs and patients, body weight and height were recorded, and the body mass index [=weight (kg)/(height (m)^2^)] was calculated. Body weight and height data were missing for one control. Symptom severity in patients was assessed with the modified Japanese Orthopedics Association (mJOA) score (19).

### 2.2. Standard protocol approval, registration, and patient consent

This prospective study was approved by the local ethics committee (Kantonale Ethikkommission Zurich, KEK-ZH 2012-0343, BASEC Nr. PB_2016-00623) and registered (clinicaltrials.gov; NCT 02170155). The study has been carried out in accordance with the Code of Ethics of the World Medical Association (Declaration of Helsinki) for experiments involving humans. Informed consent was provided by all participants prior to study enrollment. Study data were collected and managed using REDCap electronic data capture tools hosted at Balgrist University Hospital, Zurich, Switzerland (20).

### 2.3. Imaging

All patients underwent a 3 Tesla MRI scan (MAGNETOM Skyra Fit and MAGNETOM Prisma; Siemens Healthcare, Germany, Erlangen), including sagittal and axial T2-weighted (T2-w) MRI. Spinal cord motion was assessed with sagittal PC-MRI as described previously (14, 16, 18). Briefly, sagittal phase contrast measurements were placed midsagittal into the spinal cord. A predefined round-shaped region of interest (20.03 mm^2^) was centered on the spinal cord in sagittal PC-MRI at each corresponding cervical intervertebral disk level (segment C2/3–C7/T1). The velocity encoding (venc) value was set to 2 cm/s and 3 cm/s (from April 2020) based on the previous findings of cord motion (9–12, 18, 21). The velocity signal was assessed within 20 time points during a cardiac cycle. The velocity calculation was conducted as reported previously (14, 18). Cranio-caudal, anterior–posterior, and right–left oscillations were measured simultaneously. The total MRI acquisition time was ~23 min (MRI parameters are listed in Table 1). Images were processed using a free of charge, online available DICOM viewer (www.horosproject.org). In patients, cervical segments were classified as “stenotic” or “non-stenotic” for analysis (NP and MH). A segment with a loss of the CSF signal in axial T2-w imaging ventral and dorsal to the spinal cord and/or evidence of spinal cord compression was defined as “stenotic.” Segments with a visible CSF signal in axial T2-w imaging ventral and/or dorsal to the spinal cord without evidence of spinal cord compression were defined as “non-stenotic.” The number of stenotic and non-stenotic segments for each cervical level is provided in Supplementary Table 1. PC-MRI was visually checked for artifacts prior to image processing (NP and MH). MR images of one HC had to be excluded due to artifacts, and caudal cervical segments could be evaluated in 16 HCs at C6 and in 13 HCs at C7 due to the partial volume effects of the cerebrospinal fluid signal. In 71 patients with available PC-MRI in three spatial directions/planes (one patient with cranio-caudal measurement only), two MRI scans had to be excluded from analysis due to motion artifacts and one MRI scan due to aliasing artifacts in all segments. In the remaining 68 patients, no analysis was possible in one patient at C4 and C5 due to aliasing artifacts and in two patients at C4–C7 due to metal artifacts from implants. Finally, in HCs, 17 datasets at C2–C5, 16 datasets at C6, and 13 datasets at C7, and in patients, 68 datasets at C2 and C3, 65 datasets at C4 and C5, and 66 datasets at C6 and C7 were included in the study. Velocity values were calculated as described previously (14, 16, 18). PC-MRI spinal cord motion readout was the amplitude of the velocity plot during the cardiac cycle (maximum positive-to-maximum negative velocity peak). To reflect the constriction of the spinal canal, the adapted spinal canal occupation ratio (aSCOR) was calculated at each segment [aSCOR (%) = spinal cord CSA divided by spinal canal CSA multiplied by 100] (22). Axial T2-w for aSCOR calculations covered segments C2–C5 in all, segment C6 in 70, and segment C7 in 17 patients. In all HCs, all the segments were covered.

**Table 1:** Parameters of MRI sequences.

	**Axial T2w**	**Sagittal T2w**	**Sagittal PC**
TE (ms)	93	87	12.36
TR (ms)	3600	3760	60.28
Slice thickness (mm)	3	2.5	5
Flip angle (°)	150	160	10
Field of view (mm)	160	220	180
Bandwith (Hz/px)	284	260	355
Base resolution	320	384	256
Phase resolution	80%	75%	50%
Spatial resolution (mm^3^)	0.5 × 0.5 × 3.0	0.6 × 0.6 × 2.5	0.4 × 0.4 × 5.0
PAT mode	GRAPPA 2	None	None

### 2.4. Statistical analysis

Statistical analysis was conducted with SPSS (IBM SPSS Statistics for Windows, Version 28.0; Armonk, NY; IBM Corp.). Metrics were reported as group median and interquartile range (IQR). Statistical significance was set at α < 0.05. Group differences between patients and HCs were calculated using the Mann–Whitney U-test (age and BMI) and Fisher's exact test (sex). Differences between patients (subgroups stenotic and non-stenotic segments) and HCs for aSCOR and amplitude values were calculated using the Kruskal–Wallis test (at segment C2 and aSCOR at segment C7: Mann–Whitney *U*-test—only non-stenotic segments in patients). Differences between motion amplitudes in different spatial directions were calculated with the Friedman test. A Bonferroni correction for multiple comparisons was applied. Correlations between amplitude and aSCOR values were analyzed using the calculation of Spearman-rho coefficients with one-sided *p*-values.

## 3. Results

### 3.1. Subject characteristics

**Figure 1:**
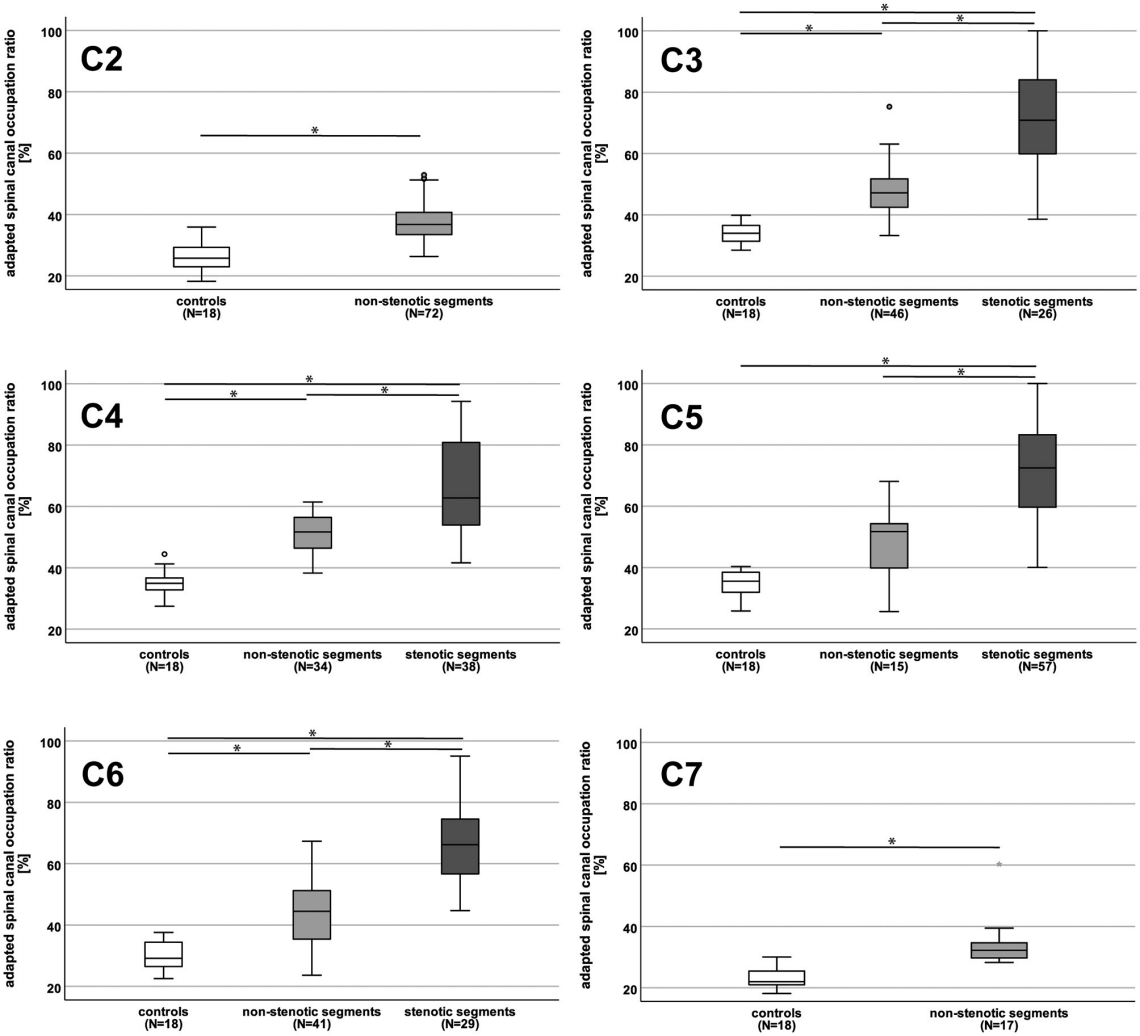
Adapted spinal canal occupation ratio in controls and patients. The adapted spinal canal occupation ratio (aSCOR; %) was higher (reflecting accentuated spinal canal constriction) in patients (light gray boxplots = non-stenotic segments; dark gray boxplots = stenotic segments) compared to controls (white boxplots) in all cervical segments, but at C5 for not-stenotic segments. In patients, stenotic segments showed higher aSCOR values compared to non-stenotic segments in all segments. At C2 no stenosis could be observed in any patient, at C7 only anatomic measurements in not-stenotic segments were available. N, number of patients ^*^p < 0.01.

HCs were older compared to patients [65.5 (57.5–67.3) versus 56.0 (47.0–65.8) years; *p* = 0.03; Table 2]. No differences were observed for BMI or sex (Table 2). The majority of DCM patients were mildly affected [mJOA score: 16 (14–18) points], and 68.1% of patients suffered from multisegmental spinal canal stenosis [number of stenotic segments: 2 (1–3)]. The spinal canal was considerably constricted (reflected by higher aSCOR values) in patients (stenotic and non-stenotic segments) compared to HCs, most severely in stenotic segments (Table 3 and Figure 1).

**Table 2:** Basic demographics of controls and patients.

	**Controls (*N =* 18)**	**Patients (*N =* 72)**	***P***
Sex (female) [%]	44.4	36.1	0.59
Age (years) [median (IQR)]	65.5 (57.5–67.3)	56.0 (47.0–65.8)	0.03
BMI (kg/m^2^) [median (IQR)]	22.8 (20.9–25.8)	24.7 (22.7–28.1)	0.08
mJOA total score (max. 18) [median (IQR)]	–	16 (14–18)	na
Multisegmental stenosis [%]	–	68.1	na
Number of stenotic segments [median (IQR)]	–	2 (1–3)	na

mJOA, modified Japanese Orthopedics Association score; na, not applicable; IQR, interquartile range.

**Table 3:** Adapted spinal canal occupation ratio values in controls and patients.

	**Controls**		**Patients non-stenotic segments**		**Patients stenotic segments**		***P***		
	***N***	**% (median [IQR])**	***N***	**% (median [IQR])**	***N***	**% (median [IQR])**	**Controls - patients non-stenotic segments**	**Controls - patients stenotic segments**	**Patients non-stenotic - stenotic segments**
C2	18	25.7 [22.8–29.8]	72	36.8 [33.4–40.9]	0	na	**< 0.01**	na	na
C3	18	34.0 [31.4–36.8]	46	47.2 [42.4–51.8]	26	70.8 [58.8–84.7]	**< 0.01**	**< 0.01**	**< 0.01**
C4	18	34.9 [32.6–36.7]	34	51.7 [46.1–56.5]	38	62.7 [53.7–81.0]	**< 0.01**	**< 0.01**	**< 0.01**
C5	18	35.6 [31.5–38.6]	15	51.7 [39.8–55.2]	57	72.5 [59.1–83.6]	0.20	**< 0.01**	**< 0.01**
C6	18	29.2 [26.2–34.6]	41	44.5 [35.2–51.3]	29	66.2 [54.8–74.6]	**< 0.01**	**< 0.01**	**< 0.01**
C7	18	22.0 [20.9–25.5]	17	32.2 [29.6–35.4]	0	na	**< 0.01**	na	na

N, number of measurements; IQR, interquartile range; na, not available. Significant findings are illustrated in bold letters.

### 3.2. Physiologic cervical spinal cord motion in HCs

**Figure 2:**
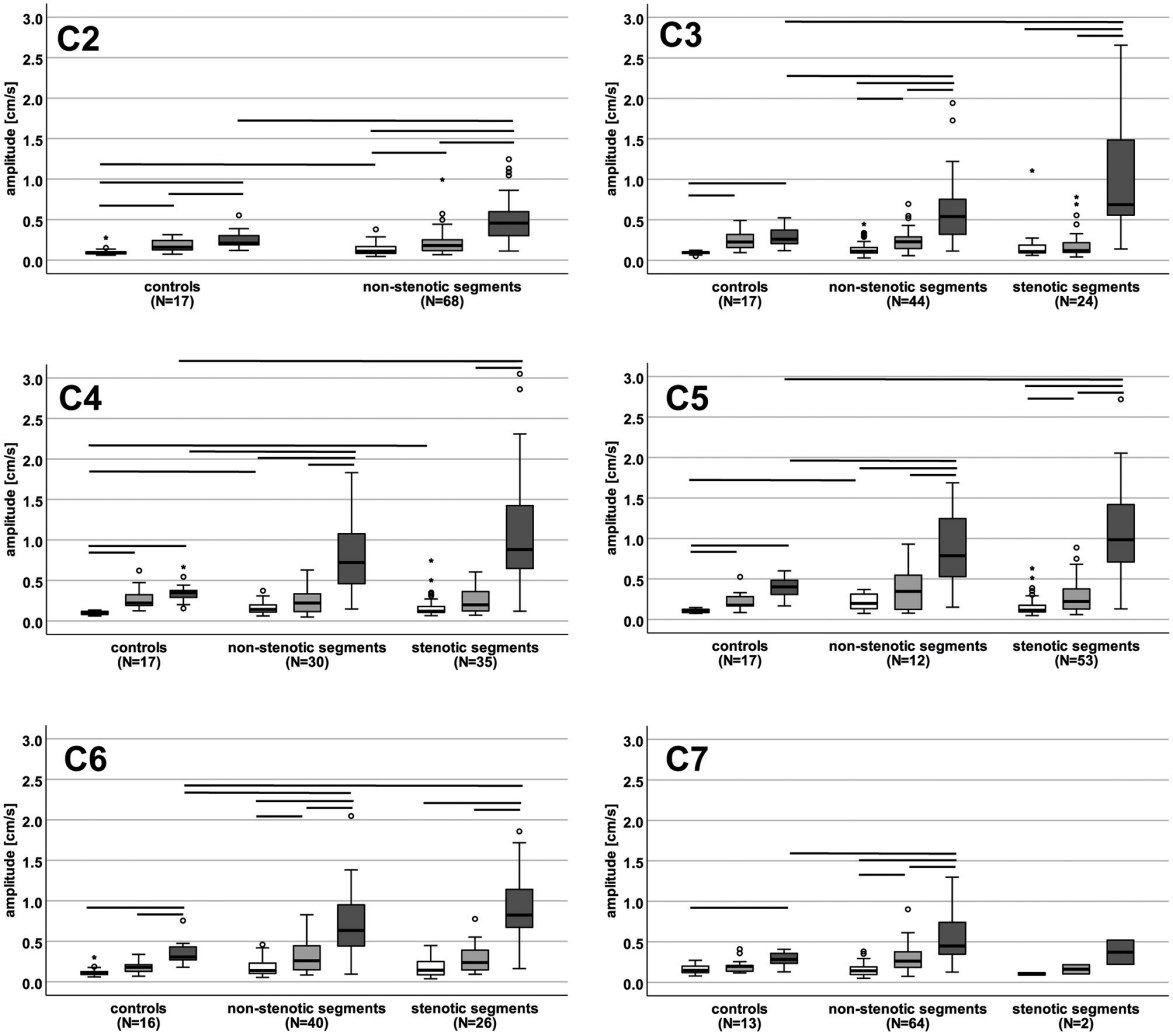
Motion amplitude values in controls and patients in different spatial directions. The boxplots show the motion amplitude values (cm/s) for right-left (white), anterior-posterior (light gray) and cranio-caudal direction (dark gray) in controls **(left)**, non-stenotic segments **(middle)** and stenotic segments **(right)** at each cervical segment. Horizontal lines represent significant differences (*p* < 0.05) between motion amplitudes within each group and between groups. N, number of patients.

Under physiological conditions in HCs, amplitude values of cervical spinal cord oscillation amplitudes were higher in the cranio-caudal direction at all cervical segments (~2–4x) and in the anterior–posterior direction at segments C2–C5 (~1.8–2.2x) compared to the right–left direction (Figure 2 and Supplementary Table 2). Amplitude values in the cranio-caudal direction were higher compared to the anterior–posterior direction at segments C2 and C6 (~1.6x; Figure 2 and Supplementary Table 2).

### 3.3. Correlations of spinal canal constriction and motion amplitudes

**Figure 3:**
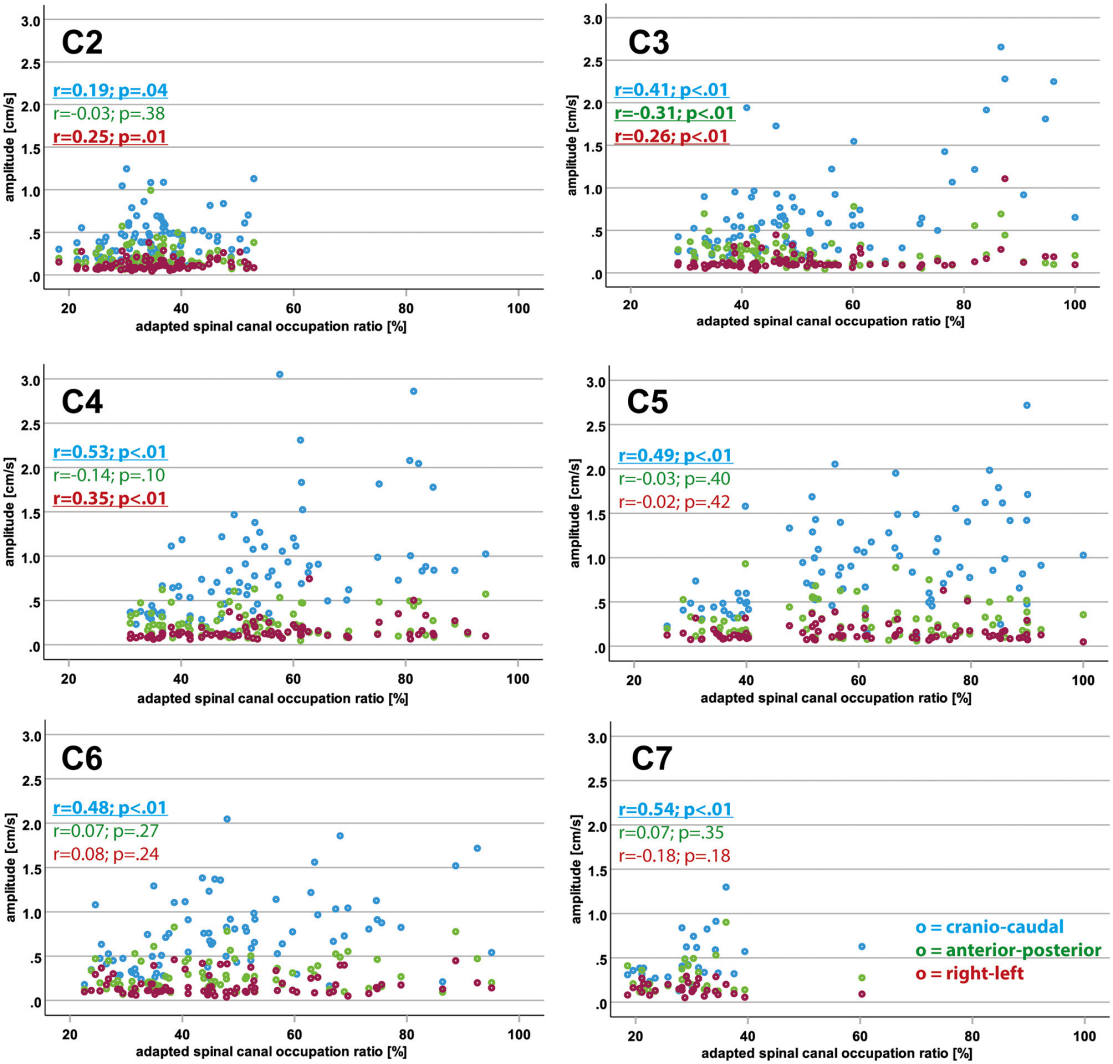
Interrelation of motion amplitude and adapted spinal canal occupation ratio values in different spatial directions. The plots show the adapted spinal canal occupation ratio values (x-axis; %) in relation to the corresponding motion amplitude values (y-axis; cm/s) for cranio-caudal (blue), anterior-posterior (green) and right-left (red) direction in the entire population (controls and patients) for each cervical segment. Significant findings for Spearman rho coefficients (r) are displayed in bold letters and underlined.

Within the entire population (controls and patients), higher aSCOR values (reflecting a more narrowed spinal canal) were correlated with higher motion amplitudes in the cranio-caudal direction at all cervical levels and in the right–left direction at C2–C4 (Table 4 and Figure 3). In contrast, amplitude values in the anterior–posterior direction became reduced with increasing aSCOR values at C3.

**Table 4:** Interrelation of adapted spinal canal occupation ratio and motion amplitude values.

**Segment**		**Cranio-caudal oscillations**		**Anterior-posterior oscillations**		**Right-left oscillations**	
	***N***	***r***	***p***	***r***	***p***	***r***	***p***
C2	85	**0.19**	**0.04**	−0.03	0.38	**0.25**	**0.01**
C3	85	**0.41**	**< 0.01**	**−0.31**	**< 0.01**	**0.26**	**0.01**
C4	82	**0.53**	**< 0.01**	−0.14	0.10	**0.35**	**< 0.01**
C5	82	**0.49**	**< 0.01**	−0.03	0.40	−0.02	0.42
C6	80	**0.48**	**< 0.01**	0.07	0.27	0.08	0.24
C7	29	**0.54**	**< 0.01**	0.07	0.35	−0.18	0.18

N, number of measurements. Significant findings are illustrated in bold letters.

### 3.4. Pathologic cervical spinal cord motion in DCM patients

In DCM patients, cranio-caudal motion amplitude values were considerably increased compared to anterior–posterior (non-stenotic segments: ~2–2.5x; stenotic segments: ~3.5–4.5x) and right–left directions (non-stenotic segments: ~3–5x; stenotic segments: ~6–9x) (Figure 2 and Supplementary Table 2). Anterior–posterior motion amplitudes in patients were increased compared to the right–left direction in non-stenotic segments at C2, C3, C6, and C7 (~1.6–2x) and in stenotic segments at C5 (~1.6–2x). No differences in the motion amplitude values could be seen between non-stenotic and stenotic segments in patients.

### 3.5. Comparison of cervical spinal cord motion between DCM patients and HCs

Compared to HCs, cranio-caudal motion amplitudes in patients showed highly increased values in non-stenotic (~2x) and stenotic segments at all cervical levels (~2.5x; except for stenotic segment C7, only two datasets were available; Figure 2 and Supplementary Table 2). In contrast, right–left motion amplitude values were only moderately increased in patients compared to HCs non-stenotic segments: C2, C4, C5 - ~1.2–2x; stenotic segments: C4 - ~1.2x). Amplitude values in the anterior–posterior direction did not differ between HCs and patients.

## 4. Discussion

**Figure 4:**
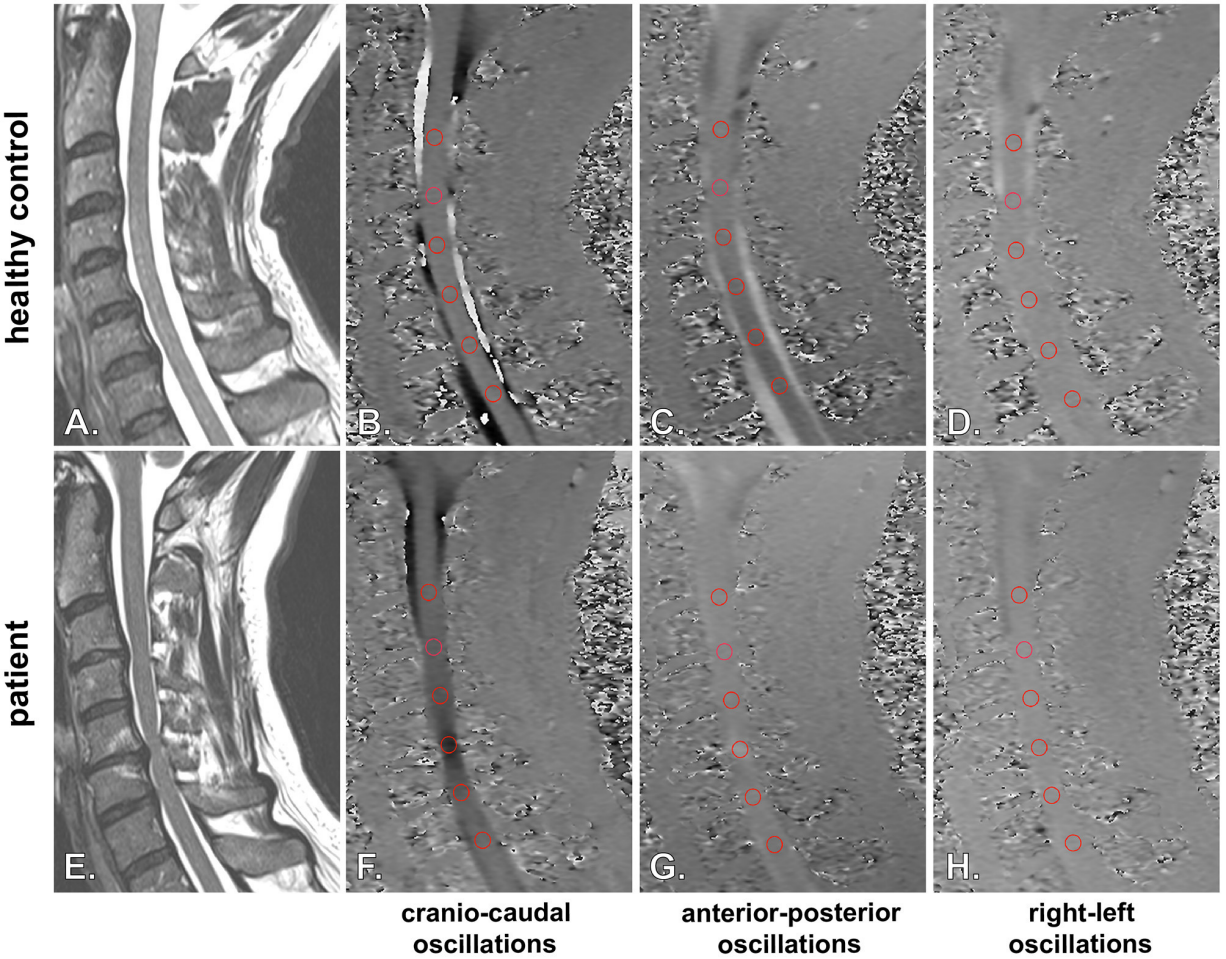
Illustration of spinal cord oscillation measurements in healthy controls and patients. Spinal cord oscillations in cranio-caudal **(B, F)** (sagittal PC-MRI), anterior-posterior **(C, G)** (sagittal PC-MRI) and right-left **(D, H**) (sagittal PC-MRI) direction are illustrated for a healthy control (**A**: sagittal T2w; **B–D**: sagittal PC-MRI) and a patient (**E**: sagittal T2w; **F–H**: sagittal PC-MRI). Higher velocities in sagittal PC-MRI are encoded with darker (**B, F**: caudal; **C, G**: posterior; **D, H**: left) respectively brighter (**B, F**: cranial; **C, G**: anterior; **D** and **H**: right) colors. Velocities were assessed with a predefined round shaped region of interest placed onto the spinal cord at the correspondend intervertebral disc level (**B–D** and **F–H**; segments C2/3-C7/T1; red circles). The representative PC-MRI pictures illustrate the highest observed velocities within the cardiac cycle. In a healthy control **(A–D**) only moderate physiologic cranio-caudal and anterior-posterior oscillations could be observed, while right-left motion was marginal. In the DCM patient extensively increased cranio-caudal oscillations were observed at the cervical stenosis at segment C5/C6 **(F)**, while anterior-posterior **(G)** and right-left oscillations **(H)** remained on low magnitudes.

This study evaluated cervical spinal cord oscillations simultaneously in all three spatial directions (i.e., cranio-caudal, anterior–posterior, and right–left direction) under physiological conditions in HCs and the pathological changes induced by spinal canal constriction in DCM patients (Figure 4). Under physiological conditions, the spinal cord was subject to cranio-caudal and anterior–posterior oscillations, while right–left oscillations were marginal. Accentuated constriction of the spinal canal was associated with increased motion amplitudes in cranio-caudal and right–left directions, while anterior–posterior motion amplitudes decreased. Interestingly, absolute amplitude values of anterior–posterior and right–left spinal cord oscillations in DCM patients remained low, comparable to HCs, while considerably increased cranio-caudal oscillations represented the cardinal pathophysiologic change.

### 4.1. Physiologic cervical spinal cord motion

In line with previous reports, our healthy cohort showed physiological anterior–posterior, right–left (15), and cranio-caudal oscillations of the spinal cord (10, 21, 23–27). In addition, it could be shown that the magnitude of anterior–posterior and cranio-caudal oscillations under physiological conditions was comparable, while right–left oscillations were much lower. Under healthy conditions, the spinal cord can oscillate in all spatial directions without any anatomic restrictions. Cerebrospinal fluid (CSF) dynamics and arterial pulsations are assumed to be the sources of spinal cord motion (8, 28–31). While systolic spinal cord motion is driven by caudal CSF flow from the cranium, diastolic cranial CSF flow and the elastic properties of the spinal cord and its surroundings (32) contribute to the upward motion of the spinal cord back to its primary position. Anterior–posterior oscillations may be mainly caused by local arterial pulsations and CSF flow dynamics, resulting in forces on the front and back of the cord. Sideward forces on the spinal cord were shown to be negligible, as only marginal right–left motion amplitudes were observed in healthy controls and DCM patients. Because the MRI measurements were all collected in the supine position, we cannot necessarily assume that a similar three-dimensional motion of the spinal cord would be observed in the upright body position. Gravity may attenuate cranio-caudal oscillations in the upright position, while anterior–posterior oscillations may be relatively increased.

### 4.2. Pathologic cervical spinal cord motion in DCM patients

Increased cranio-caudal oscillations in DCM patients compared to HCs, both in stenotic and non-stenotic segments, have been previously reported (9–14, 22). This study complements those findings by showing that amplitudes in the right–left direction increase only slightly and in the anterior–posterior direction decrease with the accentuated constriction of the spinal canal (reflected by correlation analysis). However, absolute amplitude values in the right–left and anterior–posterior directions in patients were low and comparable to HCs, in contrast to considerably increased cranio-caudal oscillations. The results are partly in line with our initial hypothesis, showing an increase in motion amplitude values, particularly in the cranio-caudal direction, while anterior–posterior and right left oscillations were low. However, no difference in anterior–posterior motion amplitudes between HCs and DCM patients could be observed. Additionally, an unexpected increase of right–left oscillations in patients was associated with narrowed anatomic conditions, while we had postulated a decrease. These alterations of cervical spinal cord oscillations in DCM patients compared to HCs may most likely be attributable to narrowed anatomic conditions. In healthy conditions, the spinal cord can oscillate unhindered in all spatial directions, and anterior–posterior and right–left oscillations in DCM patients are limited due to spinal stenosis. While spinal canal constriction is often accentuated in the anterior–posterior dimension, leaving no CSF space, preserved lateral CSF is frequently observable where there is spinal stenosis. Therefore, a slight increase in right–left oscillations in patients may be attributed to the remaining lateral space within the spinal canal, allowing the spinal cord to oscillate in this direction. However, the values of right–left oscillations remained low. In conclusion, we reason that with the progressive constriction of the spinal canal, forces mainly driven by CSF dynamics can predominantly be translated into cranio-caudal spinal cord oscillations, resulting in manifold increased movement velocity amplitudes in this direction.

### 4.3. Clinical significance of spinal cord motion measurements

The pathophysiology of DCM involves immediate cord compression, spinal malalignment causing altered cord tension, impaired vascular supply, and repeated dynamic injury (33–36). DCM patients consistently exhibit increased cranio-caudal oscillations (9, 10, 12, 13, 22). Wolf et al. found that increased cranio-caudal motion at a focal cervical stenosis mechanically strains the entire cervical cord (13). A computational model also demonstrated that cranio-caudal spinal cord oscillations can contribute to spinal cord damage in DCM, similar to dynamic compression (37). Increased cranio-caudal oscillations are associated with upper limb dysesthesia (9), impaired sensory-evoked potentials (11), and decreased sensory scores (12) in DCM patients. The impact of anterior–posterior and right–left oscillations on DCM patients is understudied and requires further investigation. Future studies should explore motion readouts in all three spatial directions and their association with clinical outcomes in DCM patients. Spinal cord motion alterations have also been reported in other pathological conditions. Tethered cord patients show limited cord motion, and markedly decreased cord motion indicates a poor postoperative outcome (38, 39). Similar changes with increased spinal cord motion have been observed in Chiari malformation and Chiari-associated syringomyelia (40–43). Spontaneous intracranial hypotension has also been linked to increased oscillations (44). Spinal cord motion measurements can be conducted using PC-MRI or ultrasound at the C1/C2 level (45, 46). PC-MRI is easily incorporated into clinical MRI protocols and provides the simultaneous assessment of all cervical levels, although simplified postprocessing methods are needed for clinical implementation. Importantly, increased spinal cord oscillations may be detected in patients before irreversible spinal cord damage occurs, aiding in clinical decision-making and timely surgical intervention to prevent impairment.

### 4.4. Limitations

Only a relatively small dataset of HCs could be used for comparison to changes in DCM patients, and the analysis of physiological changes due to aging was not included. A matched analysis, controlled for age and gender, would be more appropriate for future studies. The visual dichotomization between stenotic and non-stenotic segments may be subject to bias, and the impact of physiological cervical spine curvatures (i.e., lordosis) was not assessed. Partial volume effects in phase contrast imaging, especially at the spinal cord tissue—the cerebrospinal fluid border in spinal cord motion measurements—have to be carefully considered to avoid measurement errors. While the used region of interest for our PC-MRI analysis sufficiently covered the spinal cord size in anterior-posterior and cranio-caudal direction, in healthy controls 1 measurement at C6 and 4 measurements at C7 had to be excluded due to partial volume effects with CSF in right-left direction. Additionally, intravoxel phase dispersion may cause a certain amount of measurement error. A previous study showed no differences in spinal cord motion within different regions of the spinal cord in axial PC-MRI (14) and also demonstrated good test–retest reliability for sagittal phase contrast measurements of spinal cord oscillations (13). Therefore, measurement error due to intravoxel phase dispersion appears to be negligible.

## 5. Conclusion

Under physiological conditions, the spinal cord oscillates in cranio-caudal and anterior–posterior directions with low magnitudes. In contrast, in DCM, pathophysiological changes in spinal cord motion are transduced to manifold increased oscillations in the cranio-caudal direction, while anterior–posterior and right–left oscillations remained low in magnitude. In conclusion, this study further demonstrates cranio-caudal spinal cord oscillations as the cardinal pathophysiologic change in DCM. Further studies are warranted to prove spinal cord oscillations as a relevant biomarker reflecting dynamic mechanical cord stress in DCM patients.

## Data availability statement

The raw data supporting the conclusions of this article will be made available by the authors, without undue reservation.

## Ethics statement

The studies involving human participants were reviewed and approved by the Kantonale Ethikkommission Zurich (KEK-ZH 2012–0343, BASEC Nr. PB_2016-00623). The patients/participants provided their written informed consent to participate in this study.

## Author contributions

NP: acquisition, analysis and interpretation of data, and critical revision of manuscript for intellectual content. JR, CZ, SF, and MS: acquisition, interpretation of data, and critical revision of manuscript for intellectual content. RS: technical support, interpretation of data, and critical revision of manuscript for intellectual content. MK: technical support and critical revision of manuscript for intellectual content. JS and MB: acquisition and critical revision of manuscript for intellectual content. PF: acquisition, interpretation of data, and critical revision of manuscript for intellectual content. MF: study concept and design, interpretation of data, and critical revision of manuscript for intellectual content. AC: study concept and design, interpretation of data, and critical revision of manuscript for intellectual content. MH: study concept and design, acquisition, analysis and interpretation of data, statistical analysis, and writing the manuscript. All authors contributed to the article and approved the submitted version.

## Supplementary material

Supplementary Table 1 Number of non-stenotic and stenotic segments in patients.

Supplementary Table 2 Comparison of spinal cord motion amplitude values in different spatial directions in controls and patients.
